# Supplementary material for: Combinatorial discovery of microtopographical landscapes that resist biofilm formation through quorum sensing mediated autolubrication
Source: Nat Commun. 2025 Jun 18;16:5295. doi: 10.1038/s41467-025-60567-x (PMC12177056; doi:10.1038/s41467-025-60567-x)
Supplement: Supplementary file 1 — Supplementary Information [file 41467_2025_60567_MOESM1_ESM.pdf]

Supplementary Information

Combinatorial discovery of microtopographical landscapes that resist biofilm formation through quorum sensing mediated autolubrication

Manuel Romero[1,2Ψ], Jeni Luckett[1], Jean-Frédéric Dubern[1,2], Grazziela P. Figueredo[3], Elizabeth Ison[1,4], Alessandro M. Carabelli[4], David J. Scurr[4], Andrew L. Hook[4], Lisa Kammerling[1], Ana C. da Silva[1,2, ΨΨ], Xuan Xue[4,ΨΨΨ], Chester Blackburn[4], Aurélie Carlier[5], Aliaksei Vasilevich[6], Phani K. Sudarsanam[6], Steven Vermeulen[5,6], David A. Winkler[7], Amir M Ghaemmaghami[1], Jan de Boer[6], Morgan R Alexander[4*] and Paul Williams[1,2*]

[1] School of Life Sciences, University of Nottingham, Nottingham, United Kingdom,
[2]National Biofilm Innovation Centre, Biodiscovery Institute, University of Nottingham, Nottingham, UK
[3] School of Computer Science, University of Nottingham, Nottingham, United Kingdom.
[4]School of Pharmacy, University of Nottingham, Nottingham, United Kingdom.
[5]MERLN Institute for Technology-Inspired Regenerative Medicine, Maastricht University, Maastricht, The Netherlands.
[6]Department of Biomedical Engineering, Eindhoven University of Technology, Eindhoven, the Netherlands.
[7]Department of Biochemistry and Genetics, La Trobe Institute for Molecular Science, La Trobe University, Melbourne, Victoria 3086, Australia

*corresponding authors; email: morgan.alexander@nottingham.ac.uk; paul.williams@nottingham.ac.uk

[Ψ]Present Address: Department of Microbiology, Faculty of Biology-Aquatic One Health Research Center (iARCUS), Universidade de Santiago de Compostela, Santiago de Compostela, 15782, Spain

[Ψ Ψ] Present Address: Department of Chemistry, School of Science, Xi'an Jiaotong - Liverpool University, Suzhou, 215123, P. R. China

[Ψ ΨΨ] Present Address: Department of Chemistry, School of Science, Xi'an Jiaotong - Liverpool University, Suzhou, 215123, P. R. China

Text S1. Machine Learning Data Analysis

Software used to classify mammalian cell shape (Cell Profiler) and descriptors related to inscribed circles between topographical features obtained from ImageJ were used to analyse the design images used to create the TopoUnits, providing 66 uncorrelated topographical shape descriptors that were used to train *P. aeruginosa* and *S. aureus* attachment models. The full set of topographical descriptors is listed in **Table S1**. The code used for modelling can be found at https://github.com/Biomaterials-for-Medical-Devices-AI/Helix [1]

Datasets have been pre-processed and their distribution and normality for both dependent and independent variables were accessed to support the choice of the modelling approaches adopted.

For *P. aeruginosa,* bacterial attachment to 1,852 TopoUnits were investigated and for *S aureus* 2,084 were considered (**Fig. 1**). TopoUnits were excluded from the analysis if their signal to noise ratio was lower than 2. Multiple machine learning methods, such as XGBoost, Support Vector Machines, Linear Regression, Random Forest and Multiple Linear Regression with Expectation Maximisation (MLREM)[2] were used to generate non-linear and linear relationships between the topographies and bacterial attachment, producing good models for the datasets, with consistent results across the models. Results from the best non-linear model (Random Forest) and MLREM were coupled with Shappley Additive Explanation (SHAP)[3] method for descriptor selection. Bootstrapping without replacement using 50 runs was used to assess the model robustness over multiple data samples. The models were built based on the top three most informative descriptors, common for both datasets, as identified by SHAP. All methods were implemented in Python 3.7. Random Forest from scikt-learn version 1.22.1 using default parameters was employed to generate the ML models. Seventy percent of each dataset was used to train the models, and 30% were kept aside in a test set used to determine the predictive power of the models.

Although Random Forest has produced a better non-linear fit to the data (with average R^2 = 0.85±0.001 and average RMSE 0.24±0.001 log fluorescence for *P. aeruginosa;* and average R^2 = 0.81±0.001 and RMSE 0.19±0.001 log fluorescence for *S. aureus* in the test set), MLREM regression coefficients assisted informing the individual contribution of each descriptor to attachment (**Figs. 2b** and **2f**). MLREM results showed that there is also a strong linear correlation between the selected descriptors and bacterial attachment, with R^2 = 0.69±0.016 and RMSE 0.35±0.012 log fluorescence for *P. aeruginosa;* and R^2 = 0.69±0.029 and RMSE 0.26±0.13 log fluorescence for *S. aureus* in the test set.

For both datasets, results for additional models tested using the selected descriptors from SHAP are shown in **Table S2** for *S. aureus* and **Table S3** for *P. aeruginosa*.

Table S1: TopoUnit topographical surface descriptors derived from image analysis of the photo lithography design files using CellProfiler and Image J.

Surface Descriptor	Description
**Total Circle Area Scaled**	The total area of circle primitives scaled by feature area [0,1].
**Number of Colour Changes Diagonally**	Number of colour changes of the feature over the diagonal.
**Circle Area**	The sum of the area of circle primitives in pixels.
**Circle Diameter**	The sum of diameter of circle primitives in pixels.
**Number of Rectangles Scaled**	The number of rectangle primitives scaled by feature area [0,1].
**Number of Triangles Scaled**	The number of triangle primitives scaled by feature area [0,1].
**Feature Unit Cell Size**	TopoUnit cells have been fabricated using 3 unit cell sizes within which each feature is placed: 10 x 10 μm, 20 x 20 μm and 28 x 28 μm. This descriptor indicates the size of the unit cell for the TopoUnit.
**Inscribed Circle Number**	Number of inscribed circles that can be inserted between features, as determined by ImageJ (See Figure S1b).
**Inscribed Circle Radius 0.1 Percentile**	0.1 percentile values calculated for inscribed circles radii within a TopoChip.
**Inscribed Circle Radius 0.25 Percentile**	0.25 percentile values calculated for inscribed circles radii within a TopoChip.
**Inscribed Circle Radius 0.75 Percentile**	0.75 percentile values calculated for inscribed circles radii within a TopoChip.
**Inscribed Circle Radius 0.9 Percentile**	0.9 percentile values calculated for inscribed circles radii within a TopoChip.
**MAD Inscribed Circle Radius**	Median Absolute Deviation of the inscribed circle radii in the TopoChip.
**Maximum Inscribed Circle Radius**	Maximum value calculated for the inscribed circle radii in the TopoChip.
**Average Inscribed Circle Radius**	Average value calculated for the inscribed circle radii in the TopoChip.
**Median Inscribed Circle Radius**	Median value calculated for the inscribed circle radii in the TopoChip.
**Minimum Inscribed Circle Radius**	Minimum value calculated for the inscribed circle radii in the TopoChip.
**Mode Inscribed Circle Radius**	Model value calculated for the inscribed circle radii in the TopoChip.
**Std Dev Inscribed Circle Radius**	Standard deviation value calculated for the inscribed circle radii in the TopoChip.
**Rectangle Area**	The area of rectangular primitives.
**Rectangle Length**	The length of the rectangular primitives.
**Number of Rectangles**	Number of Rectangles in the TopoUnit cell
**Number of Triangles**	Number of Triangles in the TopoUnit cell
**Maximum Feature Area**	Area of the biggest feature in the TopoUnit cell.
**Average Feature Area**	Average feature area in the TopoUnit cell.
**Minimum Feature Area**	Minimum feature area in the TopoUnit cell.
**Maximum Feature Compactness**	The maximum value for compactness for the features in the TopoUnit cell. Compactness is calculated as the

	variance of the radial distance of the object's pixels from the centroid divided by the area.
**Average Feature Compactness**	The average compactness calculated for the features in the TopoUnit cell.
**Mode Feature Compactness**	The mode value for compactness for the features in the TopoUnit cell.
**Feature Compactness Percentile 0.1**	0.1 percentile values for compactness calculated for the features in the TopoUnit cell.
**Skewness Feature Compactness**	Skewness of the variance of the radial distance of the object's pixels from the centroid divided by the area.
**Variance Feature Compactness**	Variance of the feature compactness values.
**MAD Feature Eccentricity**	Median absolute deviation of the feature's eccentricity. The eccentricity of the ellipse that has the same second-moments as the region. The eccentricity is the ratio of the distance between the foci of the ellipse and its major axis length. The value is between 0 and 1. (0 and 1 are degenerate cases; an ellipse whose eccentricity is 0 is actually a circle, while an ellipse whose eccentricity is 1 is a line segment.).
**Maximum Feature Eccentricity**	Maximum value for feature eccentricity in the TopoUnit cell.
**Average Feature Eccentricity**	Average value for feature eccentricity in the TopoUnit cell.
**Median Feature Eccentricity**	Median value for feature eccentricity in the TopoUnit cell
**Feature Eccentricity Percentile 0.1**	0.1 percentile eccentricity values calculated for features in the TopoUnit cell.
**Skewness Feature Eccentricity**	Skewness of the eccentricity calculated for features in the TopoUnit cell.
**Variance Feature Eccentricity**	Variance of the feature eccentricity values.
**Maximum Feature Extent**	Maximum extent values for the features in a TopoUnit cell. Extend is the proportion of the pixels in the bounding box that are also in the region. Computed as the Area divided by the area of the bounding box.
**Average Feature Extent**	Average extent values for the features in a TopoUnit cell.
**Mode Feature Extent**	Mode extent values for the features in a TopoUnit cell.
**Feature Extent Percentile 0.1**	0.1 percentile extent values calculated for the features in a TopoUnit cell.
**Variance Feature Extent**	Variance of the extent values for the features in a TopoUnit cell.
**Maximum Feature Form Factor**	Maximum form factor value calculated for the features in the TopoUnit cell. Form factors is calculated as 4*π*Area/Perimeter^2. Equals 1 for a perfectly circular object.
**Average Feature Form Factor**	Average form factor value calculated for the features in the TopoUnit cell.
**Mode Feature Form Factor**	Mode form factor value calculated for the features in the TopoUnit cell.
**Variance Feature Form Factor**	Variance form factor value calculated for the features in the TopoUnit cell.
**MAD Feature Major Axis Length**	Median absolute deviation of the feature's major axis length. The length (in pixels) of the major axis of the ellipse that has the same normalised second central moments as the region.

**Maximum Feature Radius**	The maximum radius of the biggest feature in the TopoUnit cell. The radius is calculated as the distance of any pixel in the object to the closest pixel outside of the object. For high aspect ratio objects, this is 1/2 of the maximum width of the object.
**Average Feature Radius**	Average value calculated for the maximum radius of the features in the TopoUnit cell.
**Variance Feature Radius**	Variance value calculated for the maximum radius of the features in the TopoUnit cell.
**Variance Feature Minimum Feret Diameter**	The Feret diameter is the distance between two parallel lines tangent on either side of the object (imagine taking a calliper and measuring the object at various angles). The minimum Feret diameter is the smallest possible diameter, rotating the callipers along all possible angles.
**Maximum Feature Orientation**	Maximum orientation calculated for the features in the TopoUnit cell. Orientation is defined as the angle (in degrees ranging from -90 to 90 degrees) between the x-axis and the major axis of the ellipse that has the same second-moments as the region.
**Average Feature Orientation**	Average orientation calculated for the features in the TopoUnit cell.
**Median Feature Orientation**	Median orientation calculated for the features in the TopoUnit cell.
**Mode Feature Orientation**	Mode orientation calculated for the features in the TopoUnit cell.
**Feature Orientation Percentile 0.1**	0.1 percentile orientation values calculated for the features in the TopoUnit cell.
**Variance Feature Orientation**	Variance orientation calculated for the features in the TopoUnit cell.
**Number of Features**	Number of features inside a TopoUnit cell.
**Std Dev of Rotation**	The standard deviation (in degrees), is used to determine the rotation of the primitives when they are placed in the feature.
**Total Area Triangle**	The total area of circle primitives scaled by feature area [0,1].
**Feature Coverage**	Percentage of the total area occupied by the features in the TopoUnit.
**Total Perimeter**	Total perimeter of the features in the TopoUnit cell.
**Triangle Area**	The area of triangle primitives in the TopoUnit cell.
**Triangle Size**	Length of the shortest side of a triangle primitive in the TopoUnit cell.
**Percentage of Pixels Covered by Topographies**	Total number of pixels representing the topographies in the design in relation to the whole design image. This descriptor contains the same information as Total Area.

Table S2 - Modelling results for *S. aureus* dataset

Model	Set	MAE	R2	RMSE
Linear Model	Test	0.195 ± 0.005	0.692 ± 0.031	0.256 ± 0.013
Linear Model	Train	0.194 ± 0.002	0.692 ± 0.013	0.256 ± 0.006
Random Forest	Test	**0.148 ± 0.005**	**0.823 ± 0.015**	**0.194 ± 0.008**
Random Forest	Train	**0.124 ± 0.002**	**0.882 ± 0.004**	**0.159 ± 0.002**
SVM	Test	0.143 ± 0.004	0.832 ± 0.012	0.189 ± 0.007
SVM	Train	0.135 ± 0.002	0.851 ± 0.005	0.178 ± 0.003
XGBoost	Test	0.153 ± 0.005	0.807 ± 0.023	0.202 ± 0.010
XGBoost	Train	0.075 ± 0.002	0.953 ± 0.002	0.100 ± 0.002

Table S3 - Modelling results for *P. aeruginosa* dataset

Model	Set	MAE	R2	RMSE
Linear Model	Test	0.260 ± 0.009	0.704 ± 0.017	0.341 ± 0.013
Linear Model	Train	0.257 ± 0.004	0.709 ± 0.007	0.338 ± 0.006
Random Forest	Test	0.168 ± 0.005	0.858 ± 0.012	0.236 ± 0.010
Random Forest	Train	0.136 ± 0.002	0.918 ± 0.003	0.180 ± 0.003
SVM	Test	0.163 ± 0.005	0.858 ± 0.013	0.236 ± 0.012
SVM	Train	0.157 ± 0.002	0.867 ± 0.006	0.229 ± 0.005
XGBoost	Test	0.172 ± 0.005	0.848 ± 0.013	0.244 ± 0.011
XGBoost	Train	0.079 ± 0.002	0.971 ± 0.002	0.107 ± 0.003

Table S4: Bacterial strains, plasmids and primers used in this study

Strain, plasmid or primer	Genotype and/or Relevant characteristic	Source or reference
**Strain**		
P. aeruginosa		
PAO1	Wild type strain	Holloway collection
PAO1 pcE2C	PAO1 transformed with pSW002-PcE2-Crimson	This study
PAJD431	In frame deletion of *pilA* in PAO1	[4]
PAJD477	In frame deletion of *fliC* in PAO1	This study
PAO1100	In frame deletion of *wspF* in PAO1	[5]
PAJD553	In frame deletion of *rhlA* in PAO1	This study
PAJD554	In frame deletion of *rhlI* in PAO1	This study
PAJD555	In frame deletion of *rhlR* in PAO1	This study
S. aureus		
SH1000	Wild-type	[6]
Pr. mirabilis		
Hauser 1885	Wild-type	[7]
A. baumannii		
ATCC17978	Wild-type	[8]
E. coli		
DH5α	*recA1 endA1 hsdR17 supE44 thi-1 gyrA96 relA1 Δ(lacZYA-argF)U169[ϕ80 dlacZΔM15],* Nal^R	[9]
S17.1λpir	*thi pro hsdR hsdM*$^+$ *recA RP4-2-Tc::Mu-Km::Tn7 λpir*, Gm^R	[10]
**Plasmids**		
pME3087	Suicide vector for homologous recombination, ColE1 replicon, Mob; Tc^R	[11]
pEX18Gm^R	Suicide vector for homologous recombination; *oriT*$^+$, *sacB*$^+$, Gm^R.	[12]
pME6032	pVS1-p15A shuttle expression vector ; IPTG inducible ; Tc^R.	[13]
pME6032Δ*lacIQ*	pVS1-p15A shuttle expression vector containing *lacIQ* mutation ; Gm^R.	[14]
pJD112	pME3087 derivative for the generation of *pilA* in frame deletion mutant; Tc^R.	This study
pJD113	pME3087 derivative for the generation of *fliC* in frame deletion mutant; Tc^R.	This study
pEX18Δ*rhlA*	pEX18Gm^R derivative for the generation of *rhlA* in frame deletion mutant; Gm^R.	[15]
pJD115	pME3087 derivative for the generation of *rhlI* in frame deletion mutant; Tc^R.	This study
pJD116	pME3087 derivative for the generation of *rhlR* in frame deletion mutant; Tc^R.	This study
p*rhlA*	pME6032 Δ*lacIQ* derivative containing a PCR fragment of 0.888kb with *rhlA* gene; used for complementation; Gm^R.	This study

p*rhlI*	pME6032 Δ*lacIQ* derivative containing a PCR fragment of 0.666kb with *rhlI* gene; used for complementation; Tc^R.	This study
p*rhlR*	pME6032 Δ*lacIQ* derivative containing a PCR fragment of 0.726kb with *rhlR* gene; used for complementation; Ap^R.	This study
p*yedQ*	pCA24N derivative containing *yedQ* gene; Cm^R	[16]
p*pilA*	pME6032 Δ*lacIQ* derivative containing a PCR fragment of 0,450 kbp with *pilA* gene; used for complementation; Tc^R.	This study
p*fliC*	pME6032 Δ*lacIQ* derivative containing a PCR fragment of 1,467 kbp with *fliC* gene; used for complementation; Tc^R.	This study
pMMR	pME6032 derivative for tagging bacteria with mCherry	[14]
pSW002-PcE2-Crimson	Broad host-range expression vector with constitutive Pc promoter; for tagging bacteria with E2-Crimson; Tc^R.	[17]
**Primers**		
PilAΔ FW1	5′-ATATCTAGAATGCCGAACTGCTCG-3′	This study
PilAΔ RV1	5′-TTAGTTATCACAACCTTGAGCTTTCATGAATCTCTC-3′	This study
PilAΔ FW2	5′-TTCATGAAAGCTCAAGGTTGTGATAACTAAGGTGAT-3′	This study
PilAΔ RV2	5′-TATCTGCAGAAGTGGAAGTGGAGA-3′	This study
FliCΔ FW1	5′-ATATCTAGAATGCTCGAAGGCGCGCATCT-3′	This study
FliCΔ RV1	5′-TTAGCGCAGCAGGCTTGTAAGGGCCATGGTGATTTC-3′	This study
FliCΔ FW2	5′-ACCATGGCCCTTACAAGCCTGCTGCGCTAAGCCCGG-3′	This study
FliCΔ RV2	5′-TATAAGCTTAAGTCGTTCAACCCGCGCGT-3′	This study
RhlIΔ FW1	5′-ATAGAATTCTTTCCGTGGCGCGCGACCAG-3′	This study
RhlIΔ RV1	5′-ATATCTAGACAAGTCCCCGTGTCGTGCCG-3′	This study
RhlIΔ FW2	5′-ATATCTAGATACCACCCGGAATGGCTGCA-3′	This study
RhlIΔ RV2	5′-ATAAAGCTTTGGCGCTCCAGGTTGATCGA-3′	This study
RhlRΔ FW1	5′-ATAGGTACCCCTCGGCGCGCGTGGGATCT-3′	This study
RhlRΔ RV1	5′-ATATCTAGATGGCCCGGGGTATGACGCTG-3′	This study
RhlRΔ FW2	5′-ATATCTAGAATCCACCACAAGAACATCCAG-3′	This study
RhlRΔ RV2	5′-ATAAAGCTTAAGACGTCCTTGAGCAGGTA-3′	This study
RhlA Complem F	5′-TATGAATTCATGCGGCGCGAAAGTCTGTTGGT-3′	This study
RhlA Complem R	5′-TATATCGATTCAGGCGTAGCCGATGGCCATCT-3′	This study
RhlI Complem F	5′-ATACAATTGATGATCGAATTGCTCTCTGAA-3′	This study
RhlI Complem R	5′-ATACTCGAGTCACACCGCCATCGACAGCGGT-3′	This study
RhlR Complem F	5′-ATAGAATTCATGAGGAATGACGGAGGCTTT-3′	This study
RhlR Complem R	5′-ATACTCGAGTCAGATGAGACCCAGCGCCGC-3′	This study

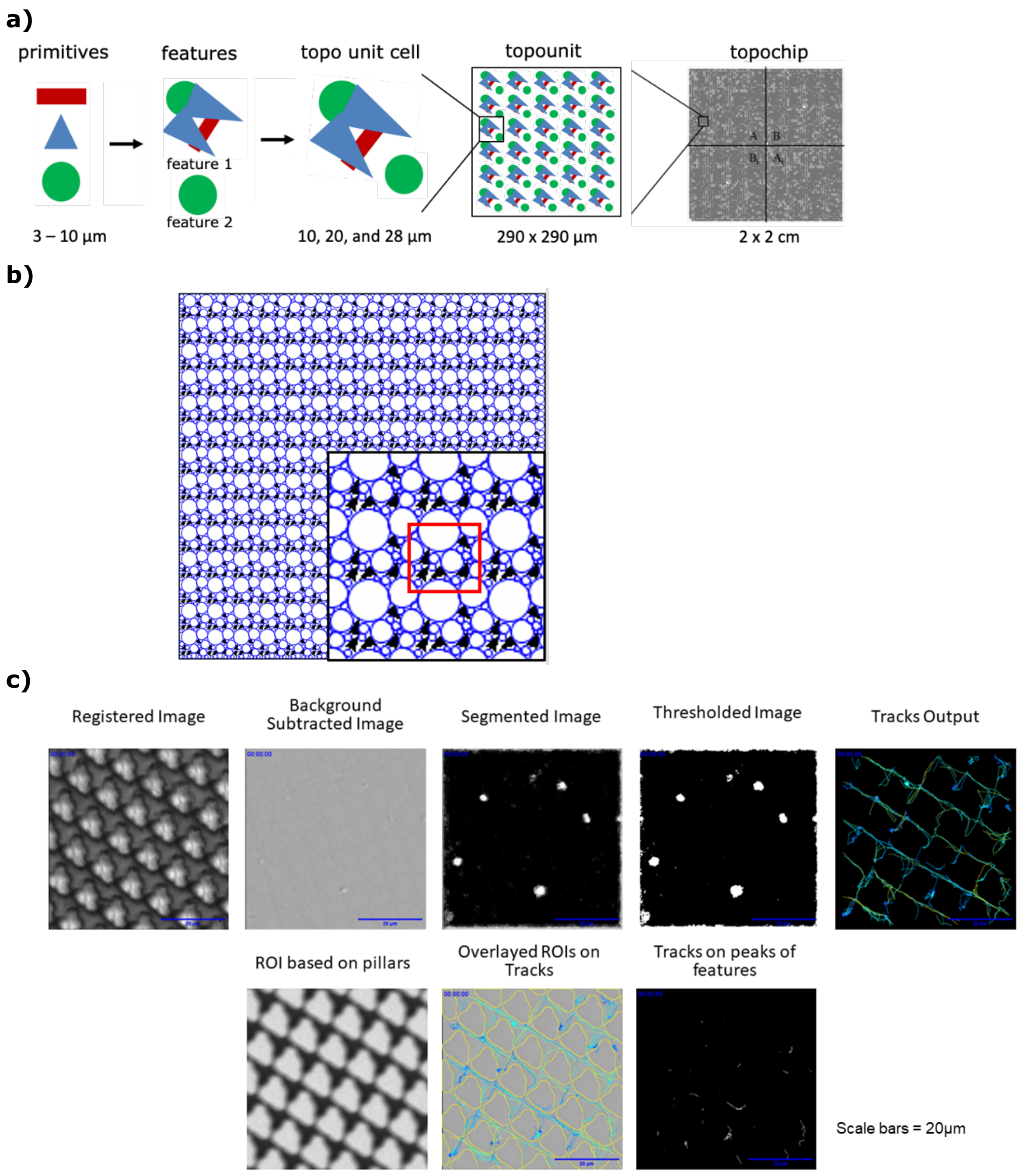

Figure S1. (**a**) Each micro-topographical element in the TopoChip contains primitives (circles, triangles and rectangles) which form 'features' that are repeated to cover the surface of a TopoUnit within a unit cell with a size of either 10 x 10, 20 x 20, or 28 x 28µm. (**b**) Image analysis of example TopoUnit, illustrating the inscribed circles (blue) used to describe the areas between the TopoUnit features (black). The unit cell is shown in red. (**c**) Representative image 'Region of interest (ROI)' maps were generated as described in the Methods section to illustrate bacterial cell tracks in relation to pillars.

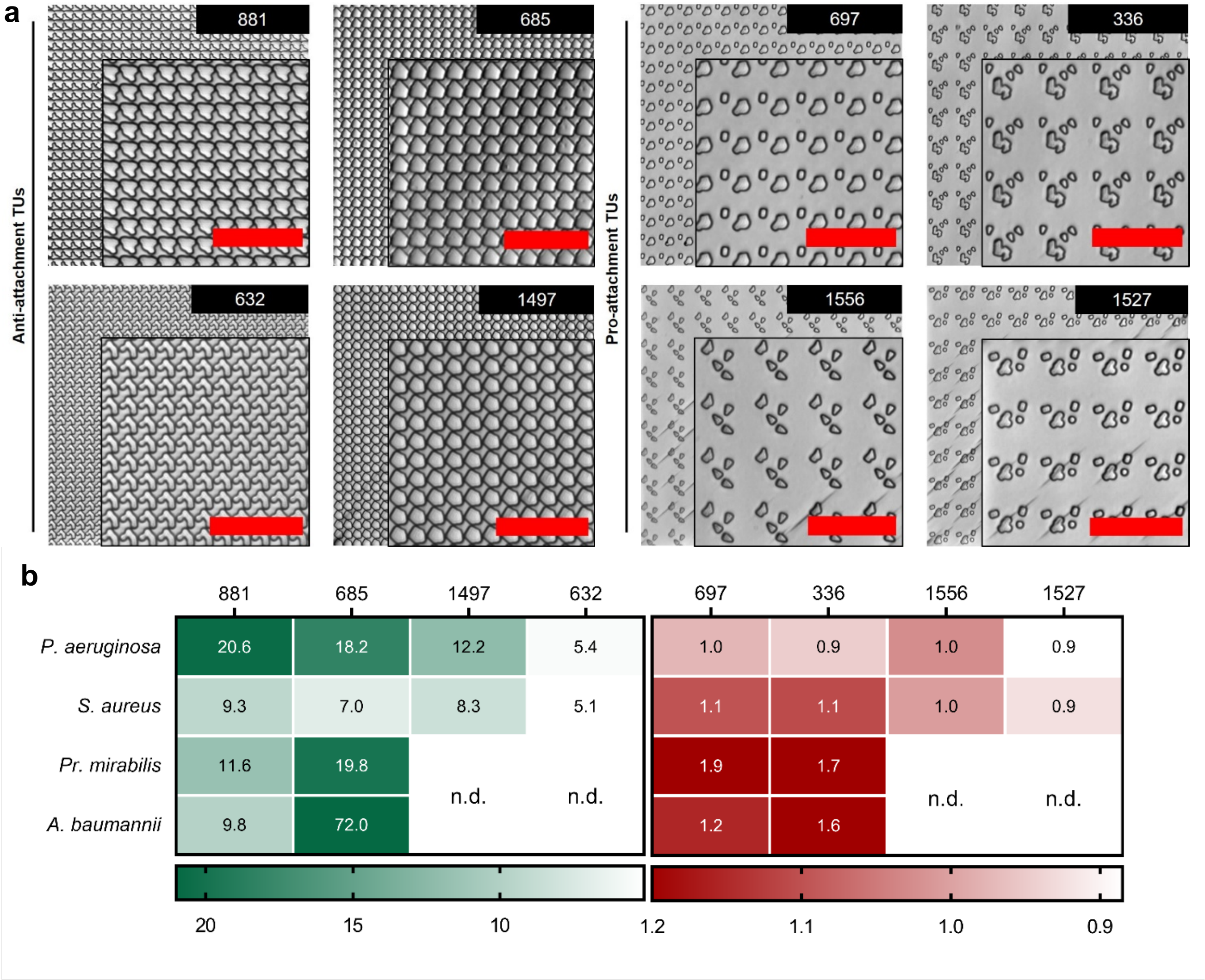

Figure S2. Selected anti- and pro-attachment micro topographies (**a**) based on the screening data obtained from quantifying *P. aeruginosa* and *S. aureus* attachment to polystyrene TopoChips. (**b**) Intensity maps of the reduction (green) or increase (red) in *P. aeruginosa*, *S. aureus*, *Pr. mirabilis* and *A. baumannii* adhesion (measured as fluorescence intensity) to selected PS TopoUnits compared with the flat control after 4 h incubation under static conditions. Scale bar: 50 μm.

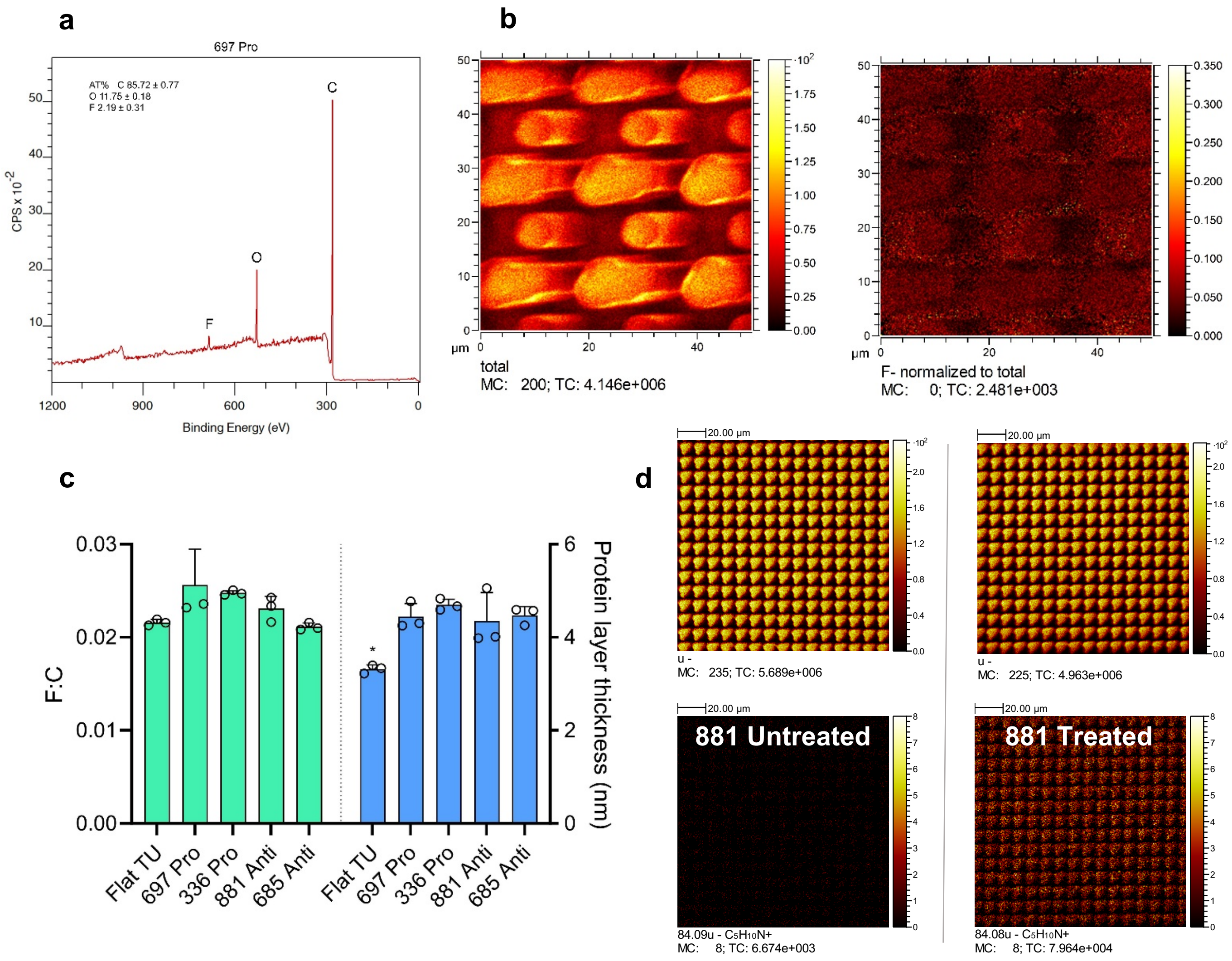

Figure S3. Polystyrene TopoChip surface chemistry analysis. (**a**) Representative XPS spectrum, obtained from a 100 × 100 µm area corresponding to a pro-attachment TopoUnit (697) in a plasma-treated chip, showing F impurity originating from the polystyrene TopoChip demoulding procedure. Atomic % for C, O and F elements are shown. (**b**) ToF-SIMS total negative and normalized F polarity secondary ion images obtained from a 50 x 50 µm area corresponding to TopoUnit 697 showing no differences in F content between topographical features of the same design. (**c**) XPS calculated F:C ratios from TopoUnits (n = 3) with pro- and anti-attachment properties against bacteria compared to flat surface control in polystyrene TopoChip (green bars). Protein depth (nm) associated with pro and anti-attachment TopoUnits and flat control after conditioning in TSB HS10% cell culture medium for 4 h (blue bars). Statistical analysis was done using a one-way ANOVA with Tukey's multiple comparisons test (*$p<0.05$). P-values for Flat vs 697, 336, 881 and 685 TopoUnits are 0.011, 0.0026, 0.018 and 0.0089, respectively. The source data are provided as a Source Data file. (**d**) ToF-SIMS total positive polarity and $C_5H_{10}N^+$ (attributed to lysine) secondary ion images obtained from a 150 × 150 µm area of an example anti-attachment TopoUnit (881) prior to and post medium treatment showing no differences in protein deposition content between topographical features. ToF-SIMS total positive polarity (labelled u-).

Text S2. TopoChip surface chemistry

As surface chemistry has a profound impact on bacterial attachment, it was essential to ensure that it was consistent across all the TopoUnits. We therefore subjected the TopoChips to time-of-flight secondary ion mass spectrometry (ToF-SIMS) for molecular characterization with relatively high lateral resolution together with X-ray photoelectron spectroscopy (XPS) for quantitative elemental analysis. Both methods detected fluorine containing impurities on the array surface, with XPS providing quantification for each TopoUnit on the array, e.g. topography 697 [F] = 2.2 ± 0.3 at% (**Fig S3a**). This could be assigned to residues from a monolayer of

trichloro(1*H*,1*H*,2*H*,2*H*-perfluorooctyl)silane (FOTS) deposited on the OrmoStamp mould to facilitate moulding. The distribution of F on the TopoUnit features, side walls and valleys was found to be constant using ToF-SIMS, within the limits of the technique imposed by the artefactual distortion of the features observed in **Fig. S3b**. Presenting a range of TopoUnits where the F to C ratio was quantified by XPS, **Fig. S3c** illustrates that there was no statistically significant difference between the units (one-way ANOVA, $p>0.05$). These results indicate that the surfaces used in the screening have uniform chemistry and that the bacteria-material interactions observed are specifically dependent on surface topography.

Since TSB containing 10% serum (TSB HS10%) was used to simulate *in vivo* growth for some experiments, XPS and TOF-SIMS analysis was carried out after incubation of TopoUnits in uninoculated TSB HS10% medium for 4 h (**Fig. 3c** and **3d**). No significant differences in the protein layer thicknesses were recorded between different TopoUnits (**Fig. S3c**). Higher levels of nitrogen (XPS) and $C_5H_{10}N^+$ (TOF SIMS; attributed lysine) were detected on topographically defined surfaces compared to flat controls after TSB HS10% conditioning, corresponding with an increase in protein layer thickness (**Fig. S3c** and **3d**). However, it is likely that the differences originate from the reduced sampling depth on the vertical feature sides that results in an over-estimation of protein layer thickness.

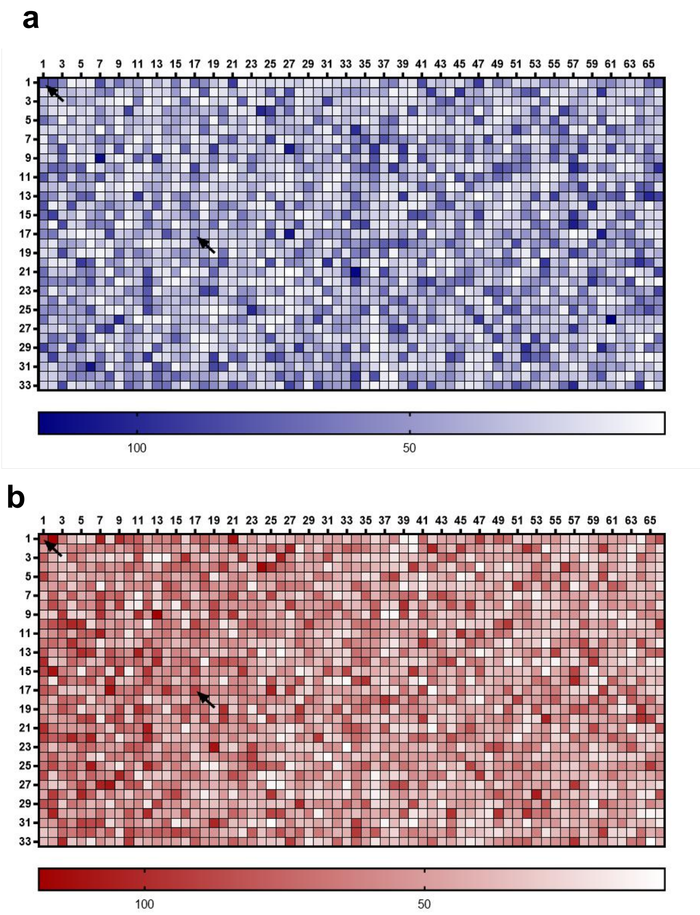

Figure S4. Intensity maps of measured fluorescence of *P. aeruginosa* (**a**) and *S. aureus* (**b**) attached to polystyrene TopoChips after 4-h incubation. Shading within each outlined square indicates the mean fluorescence intensity value for the TopoUnit (see Key at panel bottom,). Black arrows pointing towards the TopoUnit coordinates 1,1 and 17,17 indicate flat surface controls.

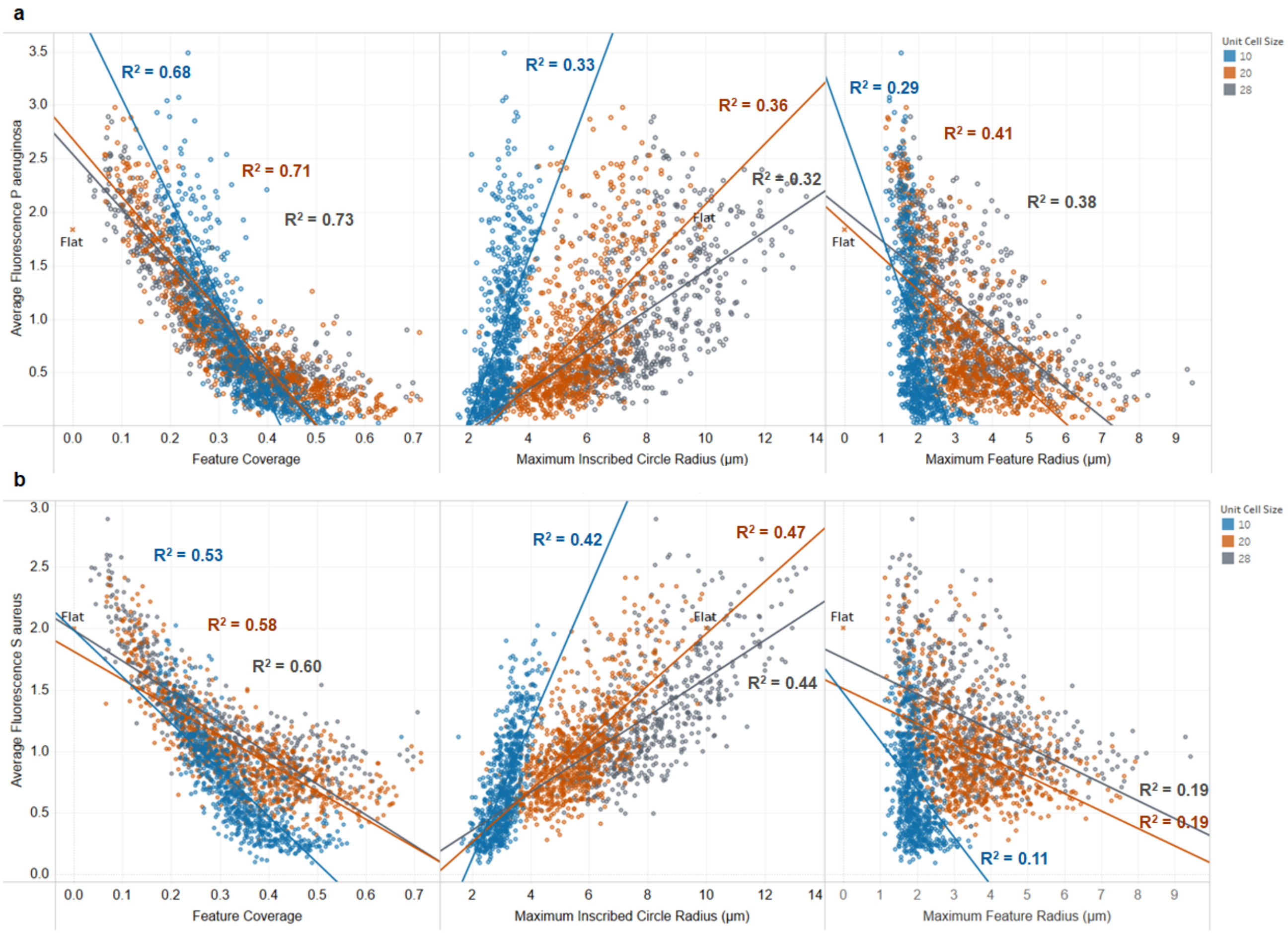

Figure S5. Topographical descriptors that show high correlation with bacterial attachment*:* (**a**) *P. aeruginosa* attachment and (**b**) *S. aureus* attachment. The topographical descriptors found to be most important for both types of bacterial attachment are the feature coverage, the maximum size of inscribed circles radii, which relate to the space between the features and the radius of the largest feature in a TopoUnit cell.

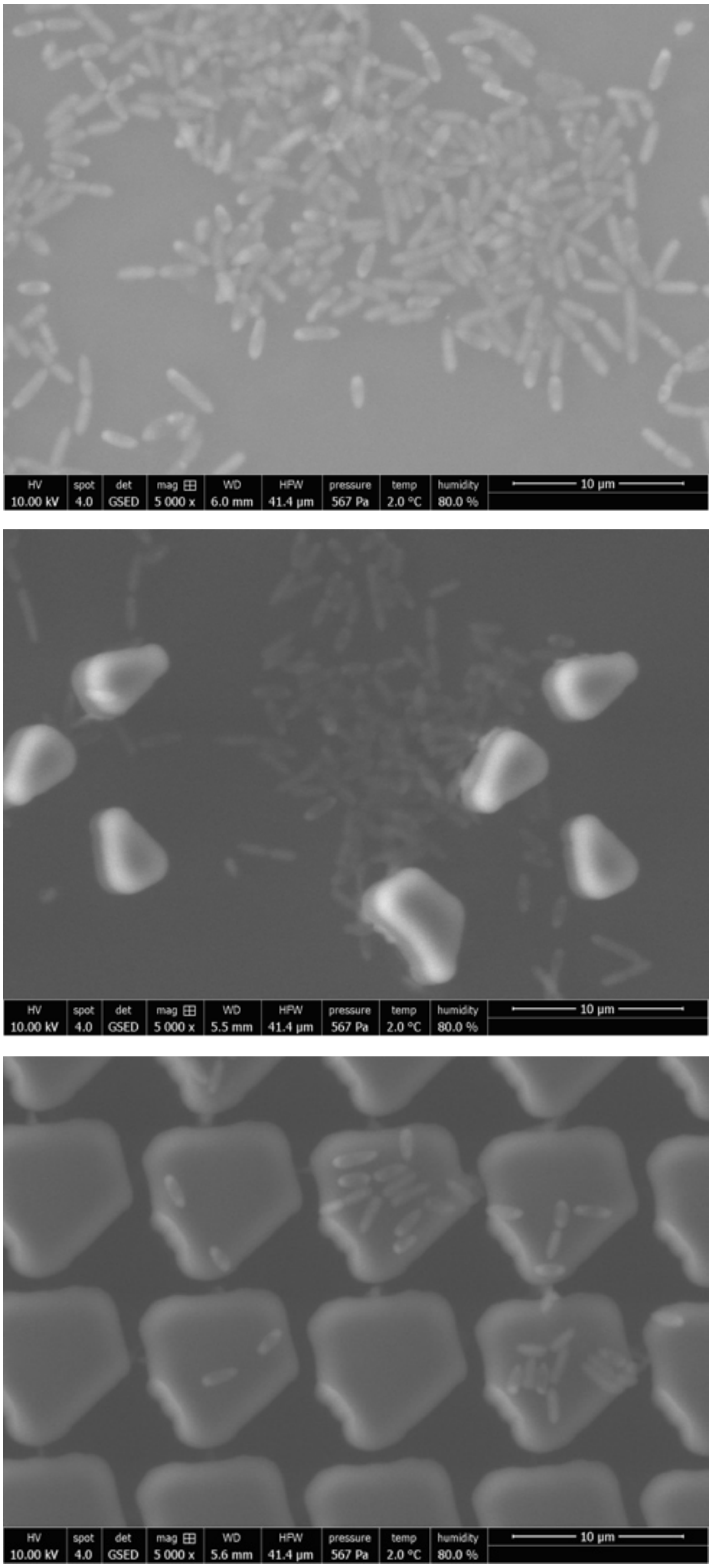

(a) Flat

(b) TopoUnit 697

(c) TopoUnit 685

Figure S6. Environmental scanning electron microscopy (ESEM) showing the morphologies of single *P. aeruginosa* cells after 4 h incubation on (**a**) flat, (**b**) pro-attachment (TopoUnit 697) and (**c**) anti-attachment (TopoUnit 685) micro-topographies after 4 h incubation. Scale bars 10 μm.

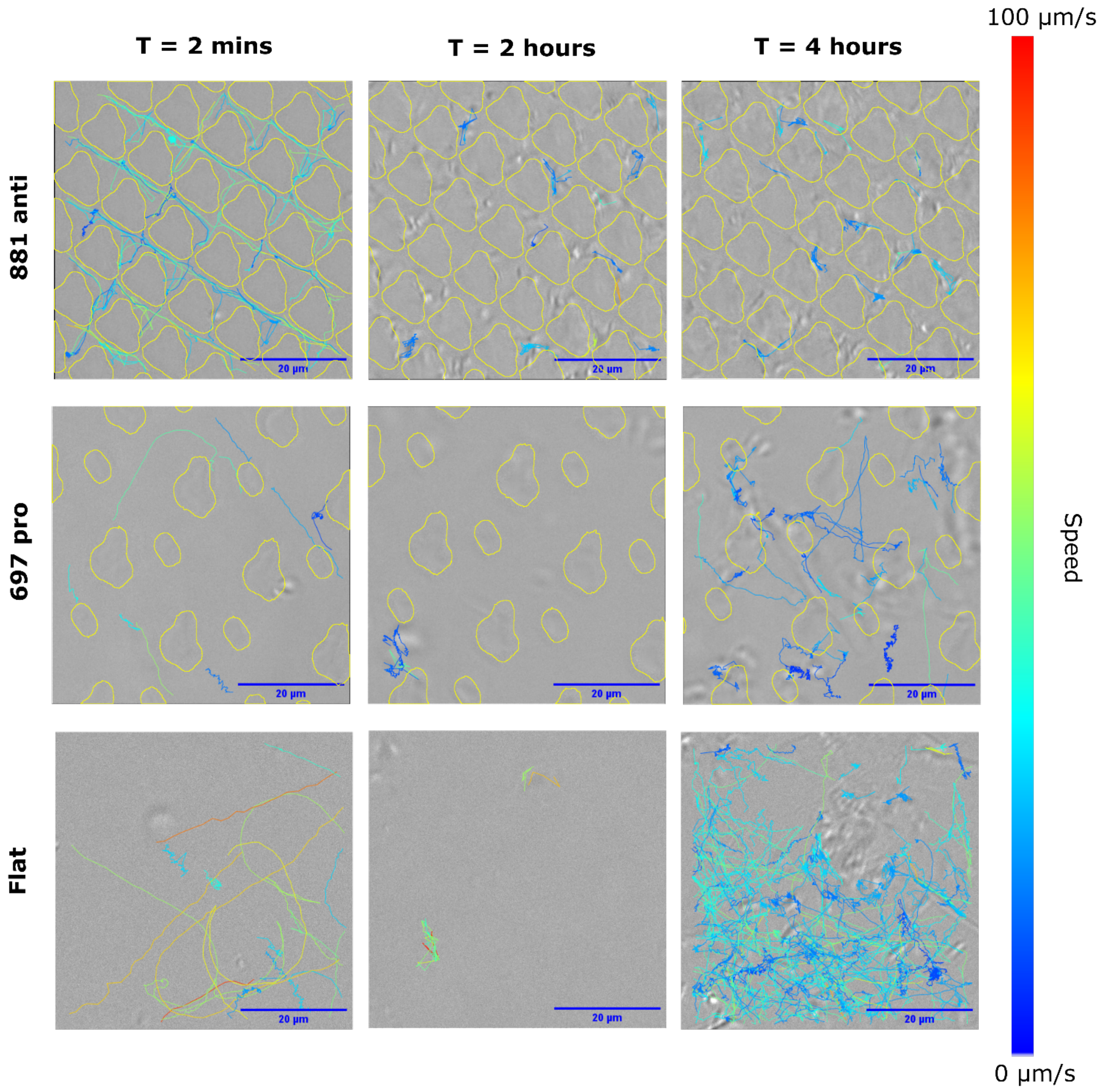
T = 2 mins
T = 2 hours
T = 4 hours
881 anti
697 pro
Flat
20 µm
100 µm/s
Speed
0 µm/s

Figure. S7 (a)

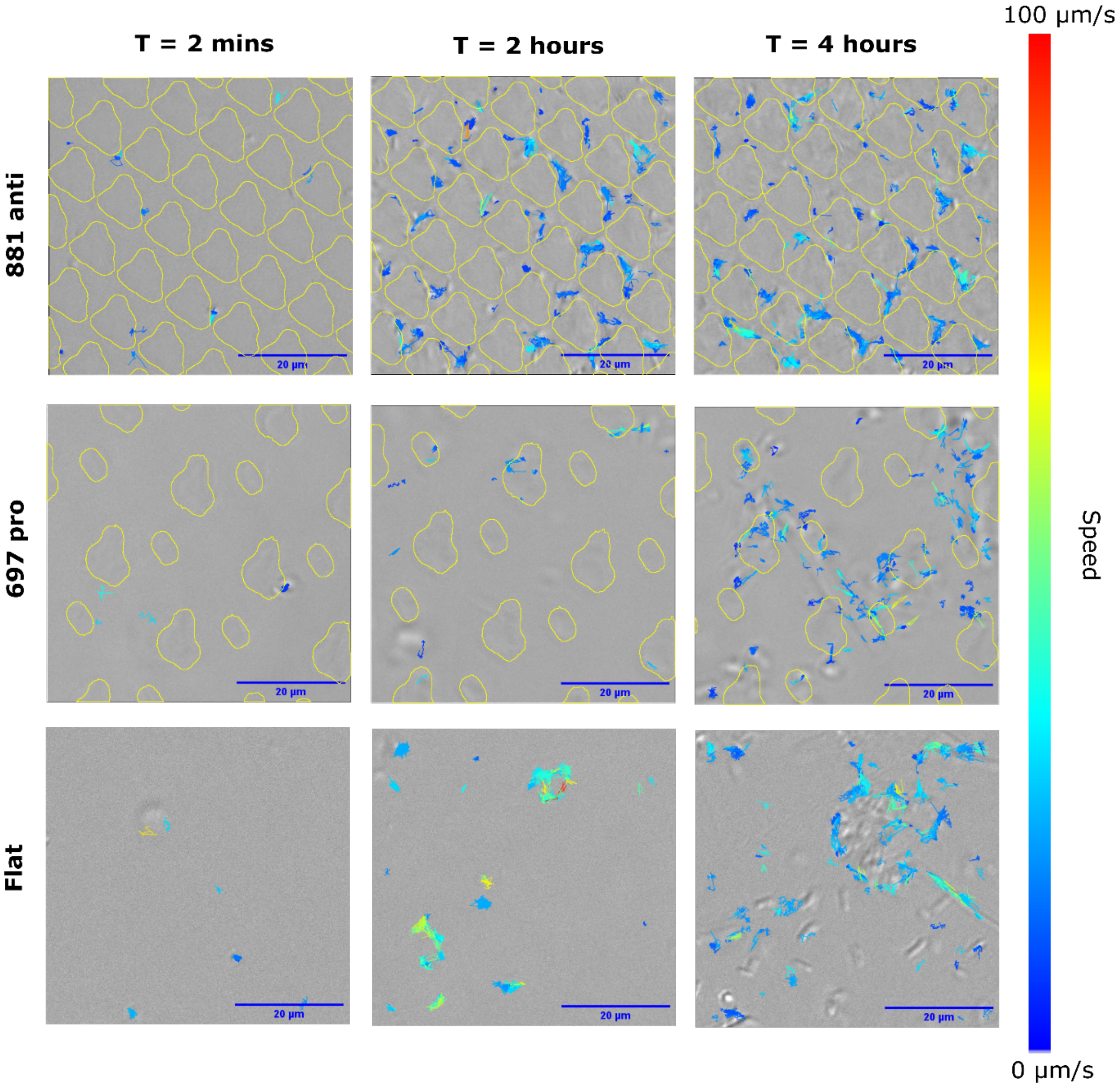
T = 2 mins
T = 2 hours
T = 4 hours
100 μm/s
881 anti
697 pro
Flat
20 μm
Speed
0 μm/s

Figure. S7 (b)

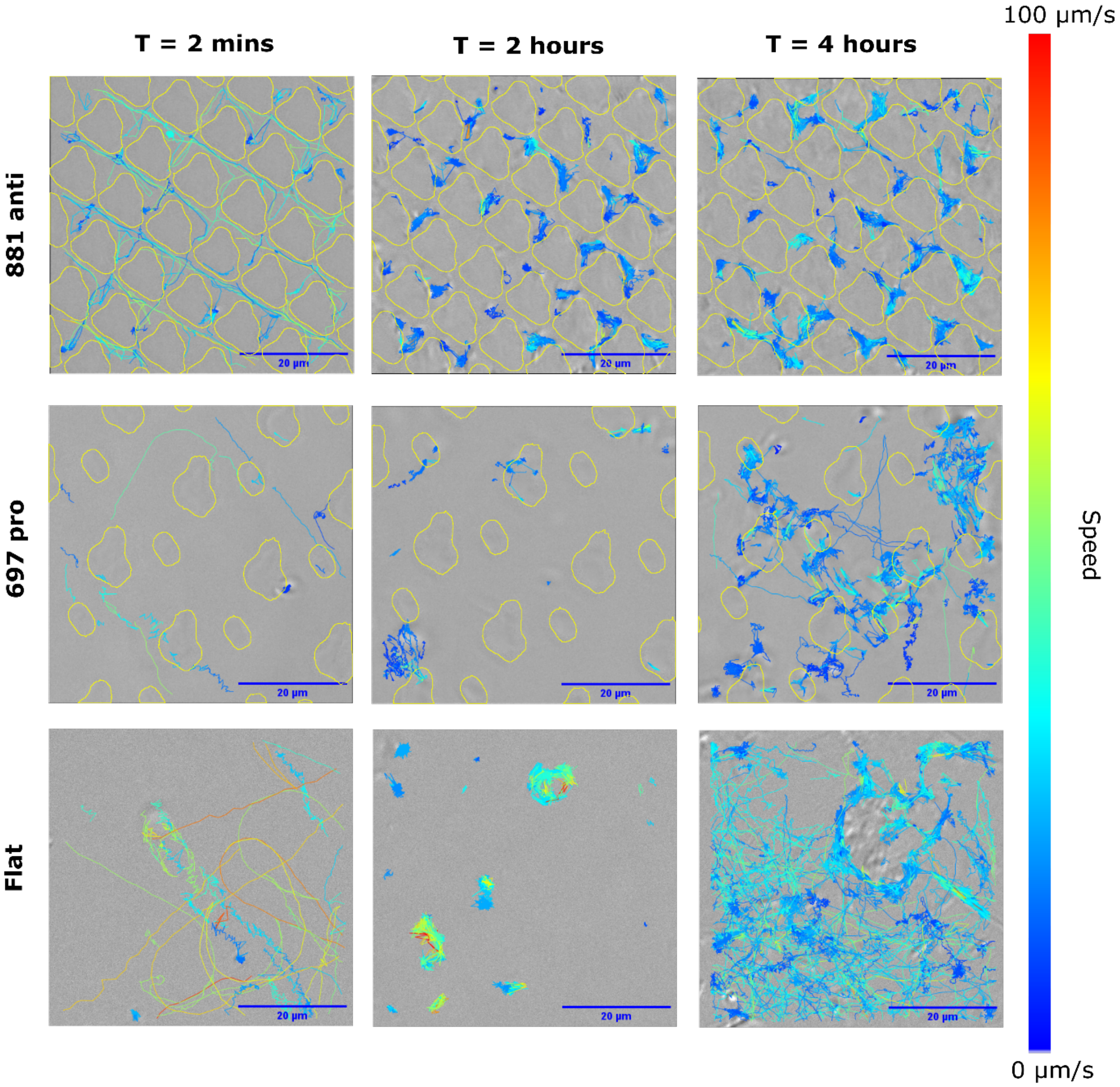

Figure S7 (c)

Figure S7. Differential interference contrast (DIC) microscopy of *P. aeruginosa* cells within 2 min of inoculation or tracked for 2h or 4h and converted into lines representing the path of an individual cell. Track speeds are depicted on the colour bar. Yellow lines indicate the positions of the subtracted topographical features. (**a**) *P. aeruginosa* tracks defined as swimming after 2 min, 2h and 4 h after inoculation on topographies 881 (anti-attachment), 697 (pro-attachment) and flat. Swimmers were defined by a low mean directional change, high maximum distance travelled, and high track displacement. (**b**) Tracks defined as stationary after 2 min, 2h and 4 h after inoculation on topographies. Stationary cells were defined by a high mean directional change, low maximum distance travelled, and low track displacement. (**c**) All tracks combined defined as both swimming and stationary after 2 min, 2h and 4 h after inoculation on topographies. Scale bars (20 μm). **Supplementary Movies 1-3** show the data collected over the first two min.

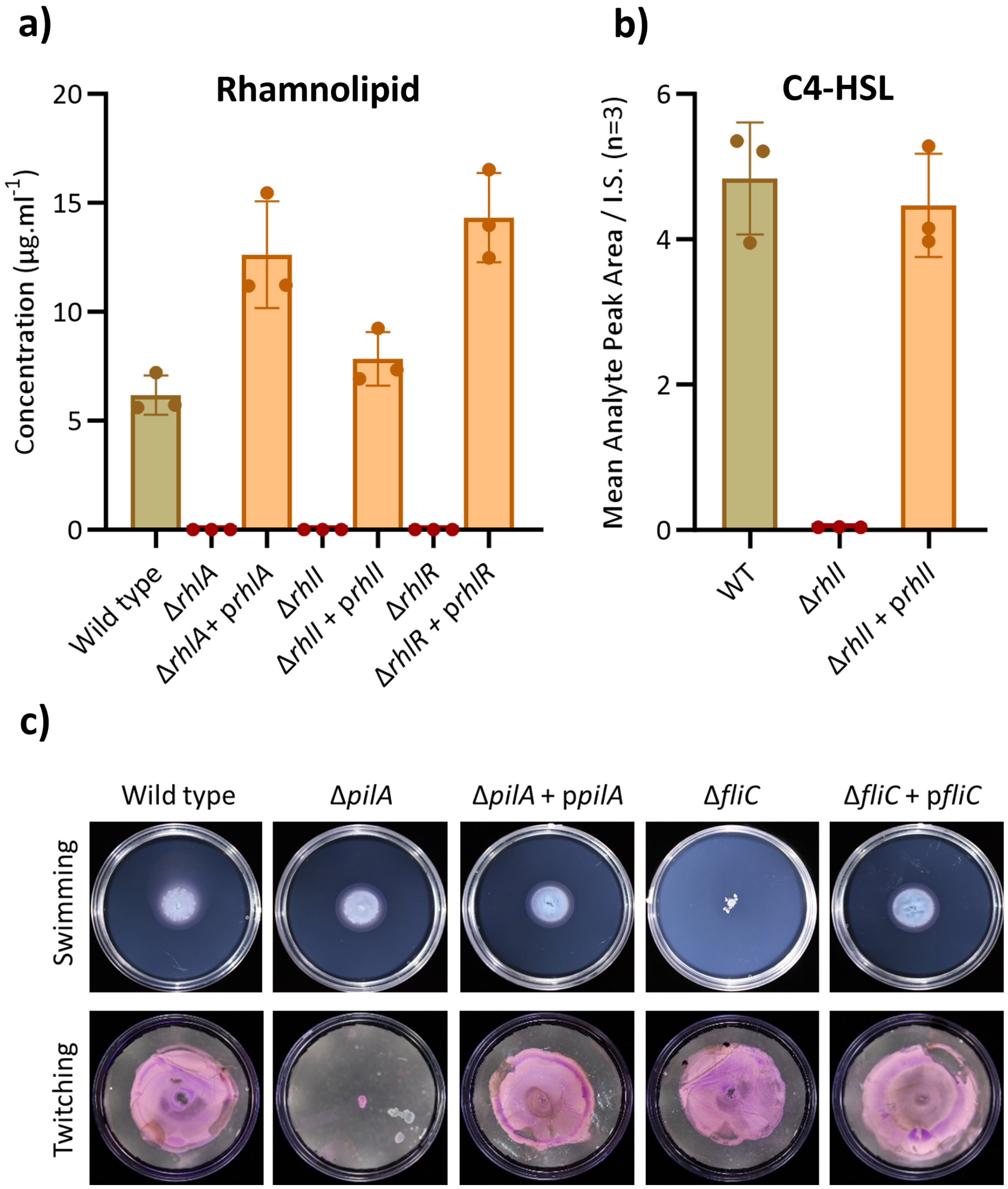

Figure S8. Rhamnolipid (**a**) and C4-HSL production (**b**) by *P. aeruginosa* PAO1 wild type, Δ*rhlA*, Δ*rhlI*, Δ*rhlR* and the corresponding mutants genetically complemented with a plasmid borne copy of the intact *rhlA*, *rhlI* or *rhlR* gene. Data shown are mean±SD; n=3; The source data are provided as a Source Data file. (**c**) Swimming, and twitching motility assays for the *P. aeruginosa* PAO1 wild type, Δ*pilA*, Δ*fliC and the corresponding mutants* genetically complemented with a plasmid borne copy of the intact *pilA* and *fliC* genes.

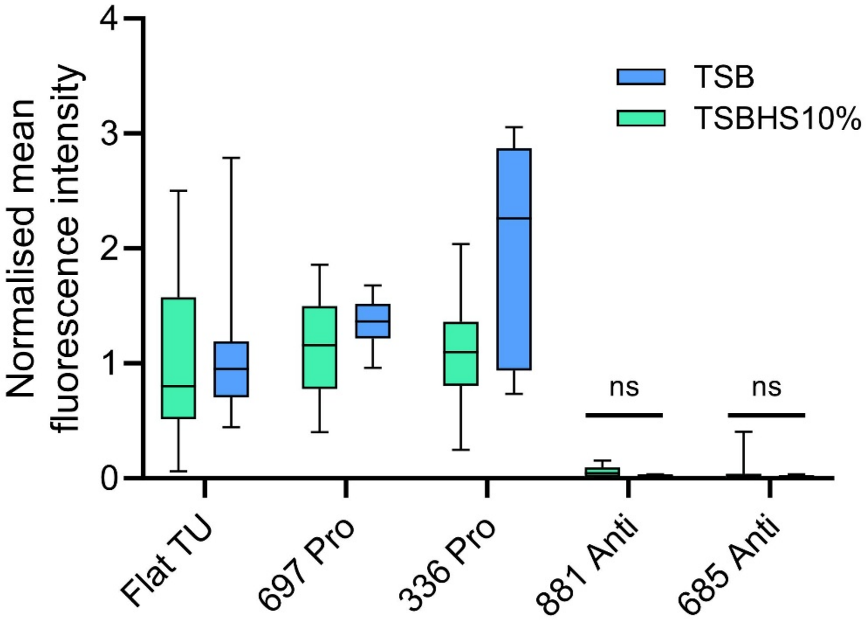

Figure S9. Comparative attachment of *P. aeruginosa* on flat, pro- (697 and 336) and anti-attachment (881 and 685) TopoUnits in TSB or TSBHS 10% after 4 h incubation under static conditions. Data shown in boxes extend from the 25th to 75th percentiles and lines in boxes correspond to the median values. Whiskers go down to the smallest and up to the largest values. Statistical analysis was done using a two-way ANOVA with Dunnett's multiple comparisons test (ns: not significant $p > 0.05$). P-values for TSB vs TSBHS10% on the 881 (n = 16 TopoUnits) and 685 (n = 22 TopoUnits) surfaces = 0.99. For Figures 4a and 4d, the source data are provided as a Source Data file.

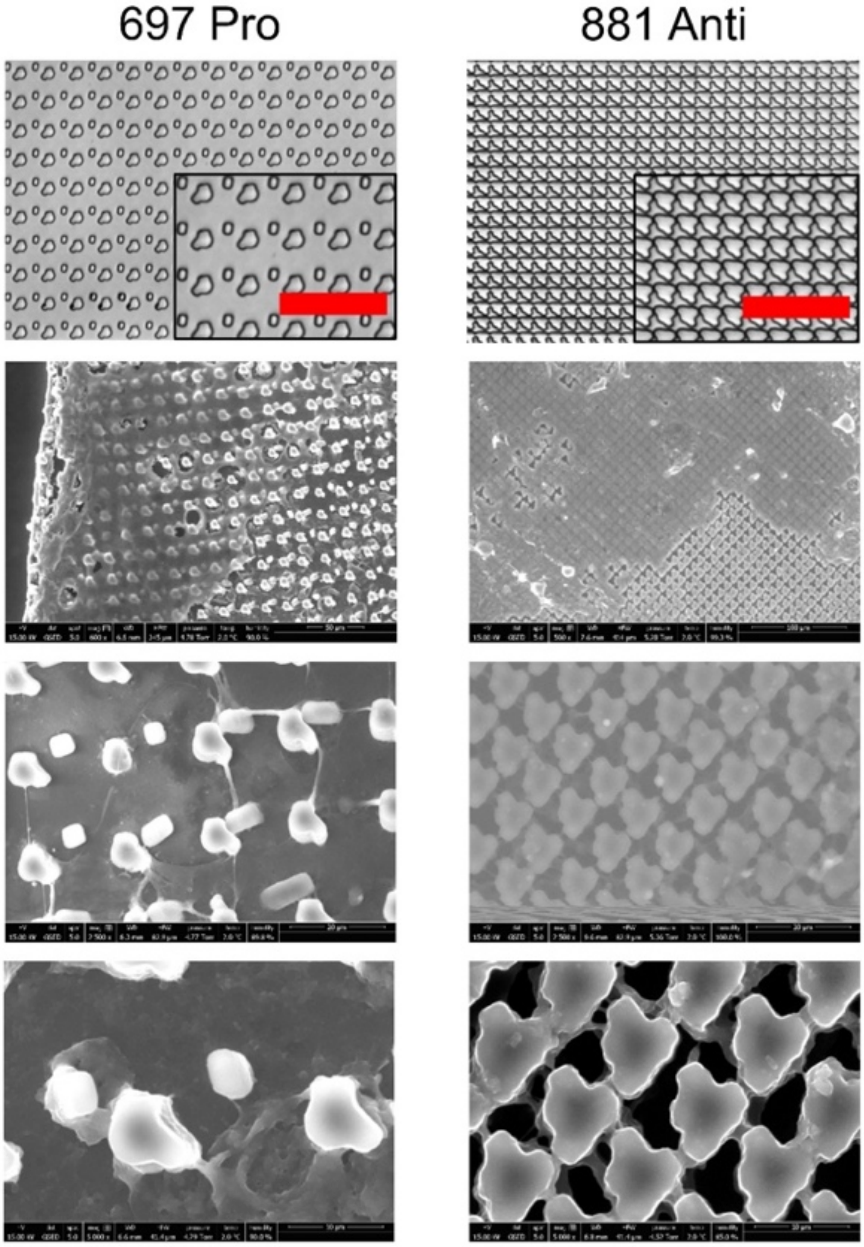

Figure S10. Representative *ex vivo* environmental scanning electron microscopy (ESEM) images of pro- (697) and anti- (881) attachment polyurethane TopoUnits removed from mice infected with *P. aeruginosa* for 4 days. Scale bar in top row bright field images: 50 µm. ESEM image scales for 697 are 50, 20 and 10 µm; for 881, they are 100, 20 and 10 µm.

REFERENCES

1. Lea, D., Mase, J.M., Aguilar, E., Omidvar, R., Figueredo, G.P., Sivikumar, K., and Mitchell-White. https://github.com/Biomaterials-for-Medical-Devices-AI/Helix.

2. Burden, F.R.; Winkler D.A. Optimum QSAR Feature Selection using Sparse Bayesian Methods. *QSAR Comb Sci*.; **28**:645-653 (2009).

3. Lundberg SM, Lee S-I. A unified approach to interpreting model predictions. In *Advances in Neural Information Processing Systems*, pp. 4765–4774. (2017).

4. Carabelli AM, Dubern J-F, Papangeli M, Farthing NE, Sanni O, Heeb S, Hook AL. Polymer-directed inhibition of reversible to irreversible attachment prevents *Pseudomonas aeruginosa* biofilm formation. *BioRxiv* doi:10.1101/2022.01.08.475475 (2022).

5. Hickman JW, Tifrea DF, Hardwood CS. A chemosensory system that regulates biofilm formation through modulation of cyclic diguanylate levels. *Proc Nat Acad USA* **102**:14422-14427 (2005).

6. Horsburgh MJ, Aish JL, White IJ, Shaw L, Lithgow JK, Foster SJ. SigmaB modulates virulence determinant expression and stress resistance: characterization of a functional *rsbU* strain derived from *Staphylococcus aureus* 8325-4. *J Bacteriol* **184**:5457–5467 (2002).

7. Hauser G. Über fäulnissbacterien und deren beziehungen zur septicämie. Ein betrag zur morphologie der spaltpilze. Leipzig, Germany: Vogel (1885).

8. Baumann P, Doudoroff M, Stanier RY. A study of the Moraxella group. II. Oxidative-negative species (genus *Acinetobacter*). *J Bacteriol* **95**:1520–1541 (1968).

9. Liss LR. New M13 host: DH5αF' competent cells. Bethesda Research Laboratories *FOCUS* **9**:3-5(1987).

10. Simon R, Priefer U, Pühler A. A Broad host range mobilization system for *in vivo* genetic engineering: transposon mutagenesis in gram-negative bacteria. *Nat Biotechnol* **1**:784–791 (1983).

11. Voisard C, Bull C, Keel C, Laville J, Maurhofer M, Schnider U, Défago G, Haas, D. Biocontrol of root diseases by *Pseudomonas fluorescens* CHA0: Current concepts and experimental approaches. In: *Molecular Ecology of Rhizosphere Microorganisms* 67-89. O'Gara F, Dowling D, Boesten B, eds. VCH Publishers, Weinheim, Germany (1994).

12. Hoang TT, Karkhoff-Schweizer RR, Kutchma AJ, Schweizer HP. A broad- host-range Flp-FRT recombination system for site-specific excision of chromosomally-located DNA sequences: application for isolation of unmarked *Pseudomonas aeruginosa* mutants. *Gene* **212**:77-86 (1998).

13. Heeb S, Blumer C, Haas D. Regulatory RNA as mediator in GacA/RsmA-dependent global 1070 control of exoproduct formation in *Pseudomonas fluorescens* CHA0. *J Bacteriol* **184**:1046-1056 (2002).

14. Popat R, Crusz SA, Messina M, Williams P, West SA, Diggle SP. Quorum-sensing and cheating in bacterial biofilms. *Proc Biol Sci* **279**:4765-4771 (2012).

15. Bukavaz S. The Effect of Rhamnolipid Production on *Pseudomonas aeruginosa* Physiology. PhD thesis. Faculty of Natural Sciences, University of Ulm, Germany (2015).

16. Kitagawa M, Ara T, Arifuzzaman M, Ioka-Nakamichi T, Inamoto E, Toyonaga H, Mori H. Complete set of ORF clones of *Escherichia coli* ASKA library (A complete set of *E. coli* K-12 ORF archive): Unique resource for biological research. *DNA Research* **12**:291-299 (2005).

17. Wilton R, Ahrendt AJ, Shinde S, Sholto-Douglas DJ, Johnson JL, Brennan MB, Kemner KM. A new suite of plasmid vectors for fluorescence-based imaging of root colonizing Pseudomonads. *Front Plant Sci* **8**:2242 (2018).
