# Supplementary material for: Combinatorial discovery of microtopographical landscapes that resist biofilm formation through quorum sensing mediated autolubrication
Source: Nat Commun. 2025 Jun 18;16:5295. doi: 10.1038/s41467-025-60567-x (PMC12177056; doi:10.1038/s41467-025-60567-x)
Supplement: Supplementary file 2 — Description of Additional Supplementary Files [file 41467_2025_60567_MOESM2_ESM.pdf]

Description of Additional Supplementary Files

File name: Supplementary Movie 1
Description: *P. aeruginosa* PAO1 movement over anti-attachment TopoUnit 881 2 min after introduction.

File name: Supplementary Movie 2
Description: P. *aeruginosa* PAO1 movement over pro-attachment 697 2 min after introduction.

File name: Supplementary Movie 3
Description: *P. aeruginosa* PAO1 movement over a flat surface 2 min after introduction.
